# Pros and Cons: High Proportion of Stromal Component Indicates Better Prognosis in Patients With Pancreatic Ductal Adenocarcinoma—A Research Based on the Evaluation of Whole-Mount Histological Slides

**DOI:** 10.3389/fonc.2020.01472

**Published:** 2020-08-21

**Authors:** Bo Li, Yang Wang, Hui Jiang, Baoming Li, Xiaohan Shi, Suizhi Gao, Canrong Ni, Zelin Zhang, Shiwei Guo, Jun Xu, Gang Jin

**Affiliations:** ^1^Department of Hepatobiliary Pancreatic Surgery, Changhai Hospital Affiliated to Navy Medical University (Second Military Medical University), Shanghai, China; ^2^Department of General Surgery, Beidaihe Rehabilitation and Recuperation Center of Joint Logistics Support Force, Qinhuangdao, China; ^3^Department of Pathology, Shuguang Hospital Affiliated to Shanghai University of Chinese Traditional Medicine, Shanghai, China; ^4^Department of Pathology, Changhai Hospital Affiliated to Navy Medical University (Second Military Medical University), Shanghai, China; ^5^Jiangsu Key Laboratory of Big Data Analysis Technique and CICAEET, Nanjing University of Information Science and Technology, Nanjing, China

**Keywords:** pancreatic ductal adenocarcinoma, prognosis, tumor–stroma ratio, whole-mount histological slides, patient stratification

## Abstract

The study aimed to investigate the potential of tumor–stroma ratio (TSR) on digitalized whole-mount histopathology to predict prognosis in patients with pancreatic ductal adenocarcinoma (PDAC). The effectiveness were evaluated through internal validation. Data were retrospectively collected from consecutive patients who underwent primary pancreatic resection from December 2016 to August 2017 (developing cohort) and from September 2017 to April 2018 (validation cohort). Digitalized whole-mount slide images were used to evaluate TSR by both pathologists and a computerized model based on Conditional Generative Adversarial Model (cGAN), respectively. TSR>1 and ≤ 1 denoted low and high stromal component. Logistic regression analysis revealed intratumoral necrosis and R1 independently associated with low stromal component in the developing cohort. Cox regression analysis revealed tumor–node–metastasis (TNM) stage [II vs. I: hazard ratio (HR), 2.584; 95% CI, 1.386–4.819; *P* = 0.003; III vs. I: HR, 4.384; 95% CI, 2.285–8.411; *P* < 0.001], stromal component (low vs. high: HR, 1.876; 95% CI, 1.227–2.870; *P* = 0.004), tumor grade (G3 vs. G1/2: HR, 2.124; 95% CI, 1.419–3.179; *P* < 0.001), and perineural invasion (with vs. without: HR, 2.147; 95% CI, 1.187–3.883; *P* = 0.011) were independent prognostic factors in the developing cohort. Stromal component categories could classify patients into subgroups within TNM stages I, II, and III based on over survival. All results were validated in the validation cohort. The weighted kappa value for categorical assessments between pathologists' evaluation and computer-aided evaluation was 0.804 (95% CI, 0.573–0.951). TSR represents a simple and reliable metric for combining the prognostic value of TNM stage in patients with PDAC.

## Introduction

Pancreatic ductal adenocarcinoma (PDAC) is the fourth leading cause of cancer-related death worldwide, with a 5-years survival rate of ~9% (1). Tumor staging systems are essential for categorizing patients into different risk groups based on prognostic factors and for guiding therapeutic approaches. However, the tumor–node–metastasis (TNM) staging system does not provide substantial predictive value. The median overall survival (OS) of patients in the same stage widely varies among different substages (2), which may be attributable to the heterogeneity of tumor cells and stroma (3). One could argue that some subpopulations could possibly benefit in terms of prognosis. To identify such potential groups, predictive parameters are necessary.

As the stroma encasing the malignant epithelial cells in pancreatic masses constitutes up to 80–90% of the tumor bulk, the stroma is now considered fundamental for tumor progression and drug delivery (4). Moreover, one recent study highlights the importance of stromal component for understanding tumor cell heterogeneity, as well as the role of these interactions in shaping tumor architecture and patient prognosis (5). Consistent with this principle, the amount of intratumoral stroma may be associated with prognosis (6). This prognostic parameter, which is also referred to as the tumor–stroma ratio (TSR), entails a simple microscopic quantification of the amount of intratumoral stroma on a tumor tissue slide, which is derived after surgical resection. Nevertheless, there exist some discrepancies in the prognostic impact of TSR in patients with PDAC. Using Masson trichrome staining or α-smooth muscle actin (α-SMA) staining in surgical specimen sections to evaluate stromal proportion, Shi et al. reported that a stromal proportion of ≤ 60% was of benefit for prognosis in PDAC (7). Heid et al. demonstrated that low tumor cellularity with a cutoff value of 30%, which was equivalent to a high amount of stroma, indicated better prognosis (8). Recently, a previous study has shown that TSR has no prognostic value in PDAC (9). The inconsistent conclusion drawn by these studies could be explained by the method for evaluating stromal proportion that only took the local part of the entire tumor into account, such as a single moderate magnification (10 ×) field with all four corners of the vision field located within the tumor (9), which was extensively used in colorectal cancer (10, 11) and other digestive tumors (12, 13). However, PDAC is characterized by a prominent feature of extensive desmoplasia (14), and evaluating the stromal proportion for the local part of the tumor may not be sufficiently accurate to estimate prognosis. Additionally, Torphy et al. analyzed the standard multiphase CT images of the whole tumor and indicated the correlation of high stromal component with favorable outcome in resected cases (15). By evaluating all tumor slides for the stromal proportion, Attiyeh et al. found that tumors with ≤ 50% stroma (*n* = 21) harbored significantly more altered genes than those with >50% stroma (*n* = 14) (16). Therefore, evaluating the stromal component for the whole tumor may clearly clarify the effect of stromal component on the prognosis of patients with PDAC.

Due to the limitations of previously used cohorts, the results of stromal component evaluation for the whole tumor were not credible enough for clinical practice. Hence, it is necessary to thoroughly investigate the predictive value of TSR for prognosis. We have routinely performed a standardized pathological examination with digitalized whole-mount slide images (DWMSIs) to facilitate TSR evaluation by semi-quantification. We hypothesized that patients with low TSR or high stromal component had better prognosis and that TSR, in addition to the TNM classification, could be a candidate marker to further stratify patients into more specific risk groups. Therefore, this study aimed to investigate the potential of TSR to predict prognosis in patients with PDAC.

## Methods

### Study Population and Data Collection

A total of 440 consecutive patients with a final histopathological diagnosis of PDAC who underwent primary pancreatic resection at the Department of Hepatobiliary Pancreatic Surgery in Changhai Hospital (Shanghai, China) were enrolled for this study. Grading and staging were performed in accordance with the WHO recommendations (17) and the 8th edition of the AJCC staging system at the time of cohort generation. Clinical and follow-up data were obtained from a prospective digital database. For each patient, the observation period started with the surgical resection. With respect to the inclusion criteria, patients who underwent (1) surgery with curative intent and (2) a standardized pathological protocol for the resected specimen were included in this study. The exclusion criteria for this study were as follows: (1) patients with intraoperative metastasis (excluded lymph node metastases) or macroscopic evidence of margin involvement (R2); (2) patients who received neoadjuvant chemotherapy or radiotherapy; (3) patients with other malignancies in the past; (4) patients who died within 90 days; and (5) patients who failed to be followed up. Subsequently, 400 patients in total were included; of these patients, 207 who underwent primary pancreatic resection from December 2016 to August 2017 composed the developing cohort and 193 who underwent primary pancreatic resection from September 2017 to April 2018 composed the validation cohort. This study was approved by the Institutional Review Board of Changhai Hospital, and no additional informed consent was required to review the patients' medical records.

### Pathological Examination

The Leeds Pathology Protocol was routinely used for pathological examination (18). The entire specimen was sliced into 5-mm-thick sections, resulting in 10–35 (average, 24.5 ± 6.7) formalin-fixed paraffin-embedded (FFPE) blocks for each specimen. Subsequently, each FFPE block was cut into 4-μm-thick sections on whole-tissue glass slides measuring 7.8 × 5.4 cm^2^. Slides stained with hematoxylin and eosin (H&E) were scanned using a Hamamatsu S60 whole slide scanner (Hamamatsu Photonics, Hamamatsu City, Japan) to obtain digitalized whole-mount slide images (DWMSIs) with an average file size of 6.47 GB (19). DWMSIs could also be observed using NanoZoomer Digital Pathology view2 software version 2.7.25. The TSR was determined in all patients with available DWMSIs. On DWMSIs in which a tumor was identified at 200 × magnification, the percentages of epithelial and stromal components were semi-quantitatively assessed using the mean value of medium power fields at 100 × magnification of the entire tumor scope on all DWMSIs (range, 2–3) of a given tumor. The TSR was estimated at 5/5, 6/4, 7/3, 8/2, 9/1, and so on. The TSR was scored independently by two senior pathologists, and any disagreement between these pathologists was resolved by discussion. We had determined “5/5 (1)” to be the best cut-off value of TSR for prognosis discrimination; hence, TSR >1 denoted low stromal component, whereas TSR ≤ 1 indicated high stromal component.

### Computer-Aided Evaluation

The DWMSIs of 41 patients from the validation cohort were used for automated TSR evaluation with each region of interest (ROI) in a DWMSI mostly included epithelium, stroma, and background/other tissues components. These components were manually delineated by pathologists for each DWMSI in the training set. The training patches were subsequently generated from each DWMSI with non-overlapping sliding windows of 512X512 pixel sliding across each ROI (19). The training patches were then fed into deep semantic segmentation model based on conditional generative adversarial networks (cGAN) (20, 21) for training. The parameters were fixed after the training procedure and then were used in the validation cohort of DWMSIs (*n* = 41). After inferencing on patches of validation cohort, the segmentation result of patches was combined in consideration of the ROI (Supplementary Figure 1). For each patient, the pixel area of the epithelium and stroma region was computed in ROI and the TSR was then be calculated. As pathological examination shown, TSR >1 denoted low stromal content, whereas TSR ≤ 1 indicated high stromal content.

### Follow-Up Protocol

The institutional follow-up was jointly completed by department follow-up specialists, and the third-party professional data were provided by LinkDoc Technology Co. Ltd. (Beijing, China). The frequency of follow-ups is done once per month during the first half year after operation, followed by once per quarter till 30th, April, 2020, the cut-off date of follow-ups in this study. The methods for follow-ups included outpatients visits, contacting by phone, mail, chatting software, or address. The general information of follow-ups included adjuvant therapy, recurrence, the cause of death, et al. The follow-up endpoint (i.e., OS) was defined as the time from operation to death. Patients who were still alive at the cut-off date of follow-ups were censored at the date at which they were last confirmed to be alive. We defined loss to follow up as no-show on the clinical follow-ups or the patients or their family members cannot be contacted by phone, mail, or address.

### Analyzed Variables

For all patients, the following demographic and clinicopathological variables were recorded in the database: sex, age, preoperative carbohydrate antigen 19-9 (CA19-9) level, tumor location (head/neck/uncinate, body/tail, or multifocal), intratumoral necrosis, perineural invasion, lymphovascular invasion, R status (R1 or R0), tumor grade (G1/2 or G3), and information on postoperative adjuvant therapy and survival time (i.e., OS). Furthermore, TNM staging was recorded according to the 8th edition of AJCC Cancer Staging Manual for Pancreatic Cancer. With respect to the tumor size, the maximum tumor diameter was reported macroscopically after microscopic corroboration had been used to place the tumors in the correct T-category according to the 8th edition of the AJCC staging system.

### Statistical Analysis

Distributional differences in baseline variables between the two cohorts and the association of TSR categories with clinicopathological features were examined using the chi-squared test or Wilcoxon rank-sum test. Variables with *P* < 0.05 in univariate analyses were included in multivariate analyses using logistic regression, and odds ratios (ORs) were calculated. Univariate and multivariate Cox regression analyses were performed to identify independent prognostic factors, and hazard ratios (HRs) were calculated. Variables with *P* < 0.1 in univariate analyses were included in multivariate analyses using a forward selection algorithm. The Kaplan–Meier method and log rank test were used to analyze “time to endpoints.” The sensitivity, specificity, positive predictive value (PPV), and negative predictive value (NPV) of the computer-aided method for TSR >1 (high stromal component) were calculated using pathologists' evaluation as the reference. The harmonic mean of recall and precision [F1 score = 2*Precision*Recall/(Precision+Recall)] was used to evaluate the accuracy of computer-aided evaluation. Agreement between pathologists' evaluation and computer-aided evaluation was measured using weighted Cohen kappa coefficient (κ). Analyses were performed using SPSS version 22.0 (IBM Corp., Armonk, NY, USA). For all analyses, a two-tailed *P* < 0.05 was considered statistically significant.

## Results

### Study Population

**Figure 1:**
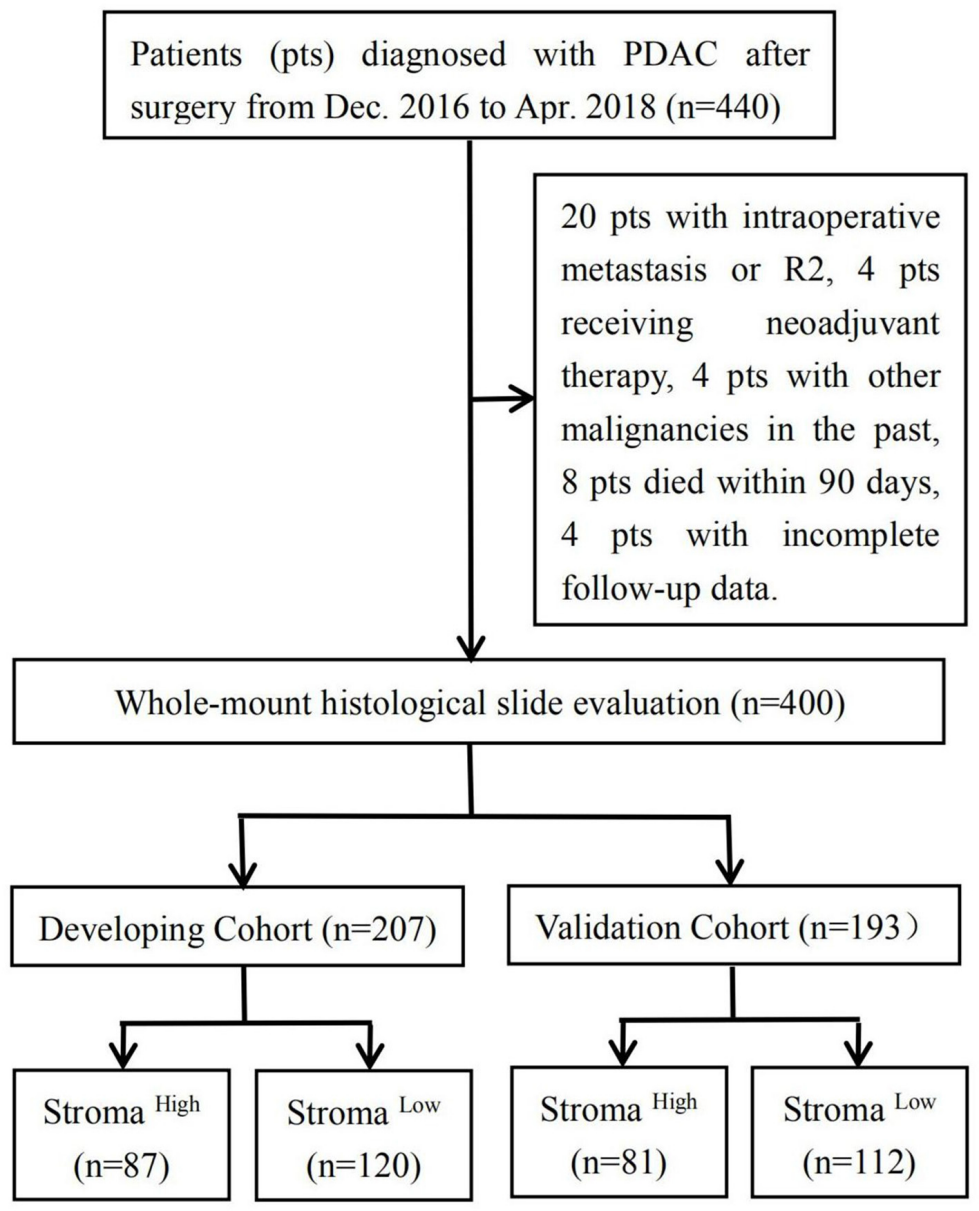
Flowchart depicting patient selection in the study.

Of the 440 consecutive patients in our study, 40 were excluded because they had intraoperative metastasis or R2 (*n* = 20), received neoadjuvant chemotherapy or radiotherapy (*n* = 4), had other malignancies in the past (*n* = 4), died within 90 days (*n* = 8), or were lost to follow-up (*n* = 4). All patients enrolled were of yellow race. The developing cohort comprised 207 patients, whereas the validation cohort consisted of 193 patients. In the developing cohort, 87 and 120 patients were deemed to have high and low stromal component, respectively; in the validation cohort, 81 and 112 patients were considered to have high and low stromal component, respectively (Figure 1). Relevant baseline variables such as age, sex, tumor location, preoperative CA19-9 level, T stage, N stage, M stage, TNM stage, tumor grade, TSR categories, R status, postoperative adjuvant therapy, and median follow-up period were similarly distributed in the developing and validation cohorts (Table 1).

**Table 1:** Baseline characteristics of patients in the developing and validation cohorts.

	**Developing cohort**	**Validation cohort**	***P***
Total	207	193	
Age, ≤ 65/>65 (years)	132/75	125/68	0.835
Sex, male/female	134/73	110/83	0.113
Tumor location, head/neck/uncinate, body/tail, or multifocal	122/75/10	109/79/5	0.365
CA19-9, <37/≥37 U/mL	58/149	45/148	0.282
*T* stage, T1/2/3	59/122/26	56/119/18	0.582
*N* stage, N0/1/2	60/99/41	61/96/43	0.456
TNM stage, I/II/III	51/112/44	57/90/46	0.319
Grade, G1/2/3	22/122/63	12/113/68	0.224
TSR, >1/≤ 1	120/87	112/81	0.849
R status, R0/R1	136/71	120/73	0.463
Postoperative adjuvant therapy, with/without	199/8	187/6	0.681
Median follow-up (months)	14.2	12.4	0.105

*CA19-9, carbohydrate antigen 19-9; TNM, tumor–node–metastasis; TSR, tumor–stroma ratio*.

### Association Between TSR Categories and Clinicopathological Variables

**Figure 2:**
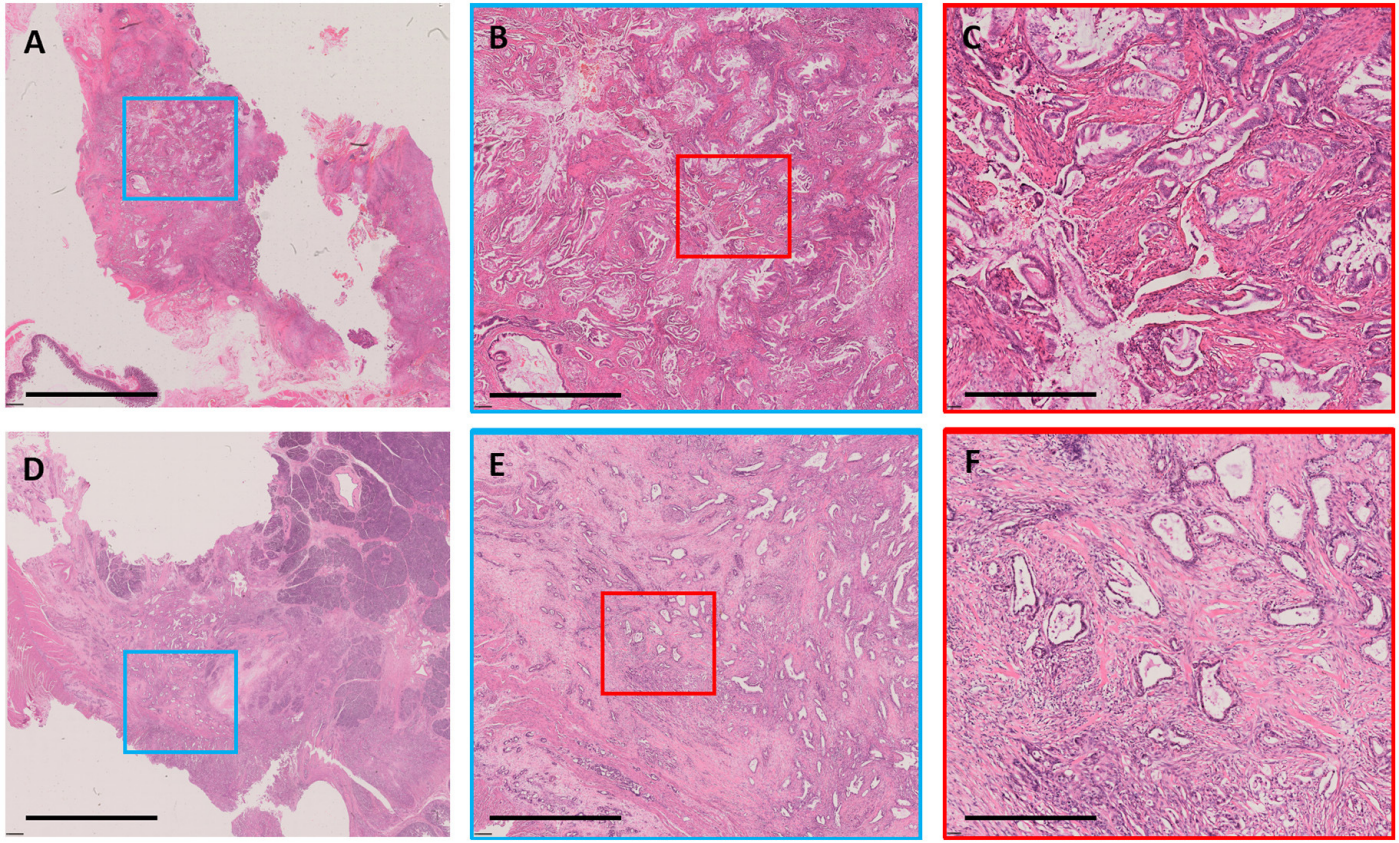
Examples of stromal component. A case with low stromal component shown as whole-mount slide image in **(A)** (3.1 ×); the blue rectangular box was amplified in **(B)** (12.5×), and the red rectangular box was amplified in **(C)** (50×). A case with high stromal component shown as whole-mount slide image in **(D)** (3.1×); the blue rectangular box was amplified in **(E)** (12.5×), and the red rectangular box was amplified in **(F)** (50×). All images were stained with H&E. The black bar represents 8 mm in **(A,D)**, 2 mm in **(B,E)**, and 0.5 mm in **(C,F)**.

Representative examples of TSR categories, including high stromal component and low stromal component, are depicted in Figure 2. Low stromal component was significantly associated with intratumoral necrosis, G3, and R1 in the developing and validation cohorts (*P* < 0.05; Table 2). In logistic regression analyses, two independent variables associated with low stromal component were identified in the developing cohort—namely, intratumoral necrosis [OR, 3.530; 95% confidence interval (CI), 1.953–6.379; *P* < 0.001] and R1 (OR, 2.281; 95% CI, 1.219–4.265; *P* = 0.01). Both variables were validated in the validation cohort (intratumoral necrosis: OR, 3.890; 95% CI, 2.097–7.217; *P* < 0.001 and R1: OR, 2.034; 95% CI, 1.059–3.910; *P* = 0.033; Table 3).

**Table 2:** Association between clinicopathological features and tumor–stroma ratio.

	**Developing cohort**		***P***	**Validation cohort**		***P***
	**High stromal component (%)**	**Low stromal component (%)**		**High stromal component (%)**	**Low stromal component (%)**	
Total	87 (42.0)	120 (58.0)		81 (42.0)	112 (58.0)	
Sex			0.478			0.560
Male	55 (63.2)	70 (58.3)		48 (59.3)	71 (63.4)	
Female	32 (36.8)	50 (41.7)		33 (40.7)	41 (36.6)	
Age (years)			0.504			0.587
≤ 65	59 (67.8)	76 (63.3)		53 (65.4)	69 (61.6)	
>65	28 (32.2)	44 (36.7)		28 (34.6)	43 (38.4)	
Tumor location			0.835			0.508
Head/neck/uncinate	53 (60.9)	71 (59.2)		41 (50.6)	66 (58.9)	
Body/tail	30 (34.5)	45 (37.5)		37 (45.7)	42 (37.5)	
Multifocal	4 (4.6)	4 (3.3)		3 (3.7)	4 (3.6)	
Intratumoral necrosis			**<0.001**			**<0.001**
Without	58 (66.7)	44 (36.7)		56 (69.1)	42 (37.5)	
With	29 (33.3)	76 (63.3)		25 (30.9)	70 (62.5)	
Grade			**0.004**			**0.011**
1/2	70 (80.5)	74 (61.7)		62 (76.5)	66 (58.9)	
3	17 (19.5)	46 (38.3)		19 (23.5)	46 (41.1)	
Lymphovascular invasion			0.185			0.809
Without	60 (69.0)	72 (60.0)		52 (64.2)	70 (62.5)	
With	27 (31.0)	48 (40.0)		29 (35.8)	42 (37.5)	
Perineural invasion			0.992			0.576
Without	16 (18.4)	22 (18.3)		10 (12.3)	17 (15.2)	
With	71 (81.6)	98 (81.7)		71 (87.7)	95 (84.8)	
*T* stage			0.234			0.198
1	30 (34.5)	29 (24.2)		29 (35.8)	27 (24.1)	
2	50 (57.5)	77 (64.2)		44 (54.3)	70 (62.5)	
3	7 (8.0)	14 (11.7)		8 (9.9)	15 (13.4)	
*N* stage			0.700			0.217
0	30 (34.5)	37 (30.8)		28 (34.6)	26 (23.2)	
1	42 (48.3)	57 (47.5)		36 (44.4)	60 (53.6)	
2	15 (17.2)	26 (21.7)		17 (21.0)	26 (23.2)	
TNM stage			0.620			0.199
I	28 (32.2)	33 (27.5)		25 (30.9)	22 (19.6)	
II	43 (49.4)	59 (49.2)		38 (46.9)	62 (55.4)	
III	16 (18.4)	28 (23.3)		18 (22.2)	28 (25.0)	
R status			**0.009**			**0.046**
0	64 (73.6)	67 (55.8)		59 (72.8)	66 (58.9)	
1	23 (26.4)	53 (44.2)		22 (27.2)	46 (41.1)	

*TNM, tumor–node–metastasis. The bold value means significant difference (P < 0.05)*.

**Table 3:** Clinicopathological features associated with low stromal component according to multivariate logistic regression analysis.

	**Developing cohort**		**Validation cohort**	
	**OR (95% CI)**	***P***	**OR (95% CI)**	***P***
Intratumoral necrosis, with vs. without	3.530 (1.953–6.379)	**<0.001**	3.890 (2.097–7.217)	**<0.001**
R status, R1 vs. R0	2.281 (1.219–4.265)	**0.01**	2.034 (1.059–3.910)	**0.033**

*CI, confidence interval; OR, odds ratio. The bold value means significant difference (P < 0.05)*.

### Prognostic Impact of TSR in Cox Regression Analysis

**Figure 3:**
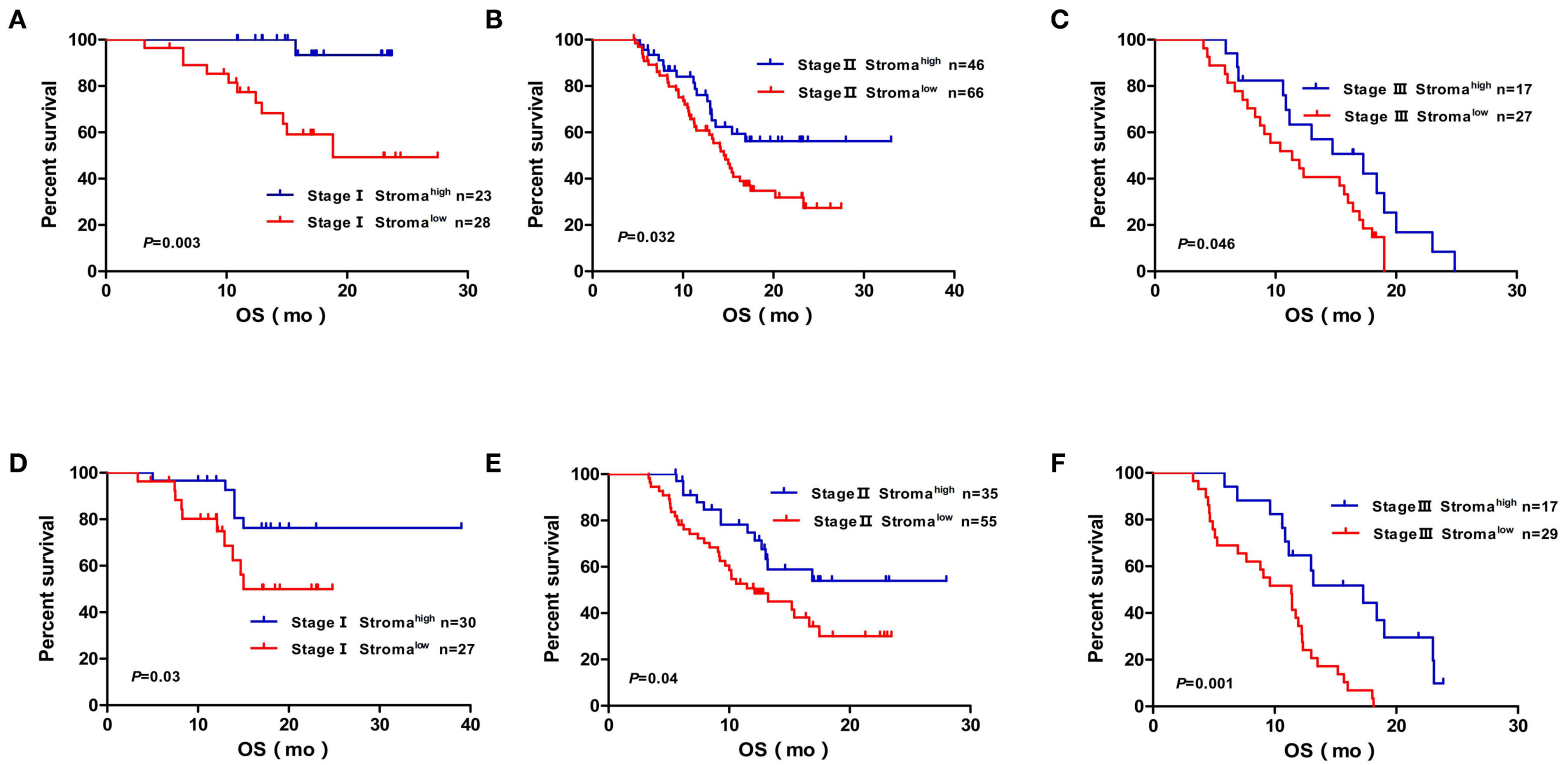
Kaplan–Meier diagrams showing OS for TSR subgroups based on the TNM stage in the developing cohort **(A,B,C)** and validation cohort **(D,E,F)**. *P*-values for log rank test are shown in each panel. OS, over survival; mo, months.

We performed Cox regression analysis to examine the effect of postoperative clinicopathological parameters on prognosis. Univariate analyses revealed that intratumoral necrosis, tumor grade, perineural invasion, T stage, N stage, TNM stage, and stromal component (low vs. high: HR, 2.094; 95% CI, 1.386–3.165; *P* < 0.001) were significantly associated with OS in the developing cohort (Table 4). Except for R status (R1 vs. R0: HR, 1.572; 95% CI, 1.062–2.326; *P* = 0.024), the analysis results of the validation cohort were almost similar to those of the developing cohort (Table 4). Furthermore, multivariate analysis confirmed that TNM stage (TNM stage II vs. I: HR, 2.584; 95% CI, 1.386–4.819; *P* = 0.003; TNM stage III vs. I: HR, 4.384; 95% CI, 2.285–8.411; *P* < 0.001), stromal component (low vs. high: HR, 1.876; 95% CI, 1.227–2.870; *P* = 0.004), tumor grade (G3 vs. G1/2: HR, 2.124; 95% CI, 1.419–3.179; *P* < 0.001), and perineural invasion (with vs. without: HR, 2.147; 95% CI, 1.187–3.883; *P* = 0.011) were independent prognostic factors in the developing cohort (Table 5). The abovementioned independent prognostic factors were also validated in the validation cohort (Table 5). Moreover, we found that stromal component categories could classify patients into subgroups and that high stromal component could predict good prognosis within TNM stages I, II, and III, which were also validated in the validation cohort (Figure 3).

**Table 4:** Univariate Cox regression analyses of clinicopathological features associated with OS of patients with PDAC.

	**Developing cohort**		**Validation cohort**	
	**HR (95% CI)**	***P***	**HR (95% CI)**	***P***
Intratumoral necrosis, with vs. without	2.021 (1.379–2.962)	**<0.001**	1.616 (1.087–2.402)	**0.018**
Grade, G3 vs. G1/2	2.303 (1.557–3.405)	**<0.001**	2.156 (1.449–3.209)	**<0.001**
Lymphovascular invasion, with vs. without	1.235 (0.842–1.811)	0.280	1.170 (0.788–1.736)	0.437
Perineural invasion, with vs. without	2.378 (1.328–4.258)	**0.004**	3.020 (1.401–6.512)	**0.005**
T stage		**0.010**		**0.022**
T2 vs. T1	1.191 (0.761–1.864)	0.443	1.510 (0.948–2.405)	0.083
T3 vs. T1	2.373 (1.322–4.262)	**0.004**	2.509 (1.300–4.841)	**0.006**
N stage		**<0.001**		**<0.001**
N1 vs. N0	1.812 (1.062–3.090)	**0.029**	1.997 (1.186–3.365)	**0.009**
N2 vs. N0	3.662 (2.082–6.439)	**<0.001**	3.915 (2.292–6.686)	**<0.001**
TNM stage		**<0.001**		**<0.001**
Stage II vs. stage I	2.641 (1.403–4.869)	**0.002**	2.545 (1.436–4.512)	**0.001**
Stage III vs. stage I	5.053 (2.643–9.660)	**<0.001**	4.707 (2.642–8.388)	**<0.001**
Stroma, low stromal component vs. high stromal component	2.094 (1.386–3.165)	**<0.001**	2.390 (1.574–3.629)	**<0.001**
R status, R1 vs. R0	1.377 (0.936–2.028)	0.105	1.572 (1.062–2.326)	**0.024**

Postoperative adjuvant therapy was not added as a variable here, because nearly all patients were treated.

*CI, confidence interval; OR, odds ratio; TNM, tumor–node–metastasis. The bold value means significant difference (P < 0.05)*.

**Table 5:** Multivariate Cox regression analyses of clinicopathological features associated with OS of patients with PDAC.

	**Developing cohort**		**Validation cohort**	
	**HR (95% CI)**	***P***	**HR (95% CI)**	***P***
TNM stage		**<0.001**		**<0.001**
Stage II vs. stage I	2.584 (1.386–4.819)	**0.003**	2.122 (1.186–3.794)	**0.011**
Stage III vs. stage I	4.384 (2.285–8.411)	**<0.001**	4.443 (2.042–6.625)	**<0.001**
Stroma Low stromal component vs. high stromal component	1.876 (1.227–2.870)	**0.004**	2.047 (1.322–3.168)	**0.001**
Grade G3 vs. G1/2	2.124 (1.419–3.179)	**<0.001**	1.751 (1.158–2.649)	**0.008**
Perineural invasion With vs. without	2.147 (1.187–3.883)	**0.011**	2.351 (1.080–5.119)	**0.031**

*CI, confidence interval; HR, hazard ratio; TNM, tumor–node–metastasis. The bold value means significant difference (P < 0.05)*.

### Agreement Between Pathologists' Evaluation and Computer-Aided Evaluation

To alleviate the pathologists' workload and facilitate standard integration of TSR into routine diagnostics, we compared the evaluation conducted by pathologists and that performed using a computer. Of the 41 patients, 20 were placed by the pathologists in the high stromal component category, whereas 21 were placed in the low stromal component category. In comparison, 18 were placed by the computer in the high stromal component category, whereas 23 were placed in the low stromal component category. After comparing the stromal component categories, the weighted kappa value for categorical assessments between the pathologists' evaluation and computer-aided evaluation was 0.804 (95% CI, 0.573–0.951), suggesting strong agreement (Table 6). With pathologists' evaluation as the reference, the sensitivity, specificity, PPV, and NPV for the classification of high stromal component by the computer-aided method were 85, 95.2, 94.4, and 87%, respectively. The precision and recall for the classification of high stromal component by the computer-aided method as compared to pathologists' evaluation were 94.4 and 85%, respectively. Furthermore, the F1 score was calculated as 89.4%, indicating the high accuracy of the computer-aided method for TSR evaluation as compared to pathologists' evaluation.

**Table 6:** Agreement between pathologists' evaluation and computer-aided evaluation and the weighted kappa value.

		**Pathologists' evaluation**		**Kappa (95% CI)**
		**High stromal component**	**Low stromal component**	
Computer-aided evaluation	High stromal component	17	1	0.804 (0.573–0.951)
	Low stromal component	3	20	

*CI, confidence interval*.

## Discussion

Based on two representative, well-characterized cohorts of 400 patients with sporadic PDAC, we first showed in our study that the application of the entire tumor scope at 100 × for TSR assessment on DWMSIs was a reliable evaluation method for classifying patients with PDAC into subgroups. Because of the intratumoral heterogeneity of PDAC, we evaluated the TSR by assessing the entire tumor scope in order to avoid selecting the most appropriate area for assessment within the tumor. However, the methods that we used might have increased the workload of pathologists, which may hamper its application in routine pathological reporting. Hence, we explored the computerized model to offset the shortcoming. The results of computerized evaluation for TSR, were highly consistent with those of pathologists' evaluation. This considerably simplifies TSR assessment and can facilitate the standard integration of TSR into routine diagnostics, promoting its regular inclusion in histopathology reports and helping in the more accurate prognostic classification of patients with PDAC. Nevertheless, there is still uncertainty about whether or not immunohistochemistry, which can distinguish activated stroma (22), should be used. Although a previous study showed that the increased α-smooth muscle antigen positive stromal component of tumor indicated the reduced survival time of patients with PDAC (23), fibroblast activation protein-α has recently rose to prominence as a marker that defines a more pro-tumorigenic stromal component (24). Thus, activated stroma evaluation may be more complicated and unreliable; in addition, the immunohistochemistry technique is more complex, expensive, and irreproducible than H&E staining and is difficult to apply in the routine pathological reporting system. Based on the above analyses, the pathological technique for TSR evaluation that we employed is fairly exact, simple, cost-effective, and reproducible to be used for classifying patients with PDAC into subgroups.

We used the optimized evaluation method to assess TSR and found that G3 was closely related to the low stromal component of the tumor, which is consistent with the finding of a previous study wherein a high degree of desmoplasia was inversely correlated to differentiated tumor grade in genetically engineered mice (22). The results of our study indicated that intratumoral necrosis and R1 were independently associated with low stromal component. Interestingly, treatment with halofuginone, which altered the immune landscape in PDAC by decreasing the stromal component, with greater immune infiltrate into low-hyaluronan regions, was reported to result in an increased number and distribution of both classically activated inflammatory macrophages and cytotoxic T cells. In concert with a direct effect on carcinoma cells, this led to widespread intratumoral necrosis (25). Hence, low stromal component and weakened stromal barriers in tumors may facilitate the infiltration of inflammatory and immune cells, which may directly participate in the formation of intratumoral necrosis. In another study, neoadjuvant treatment resulted in tumor cell death, with the remaining tumor cells lying at a greater distance from each other, which was closely related to a low R1 rate (26). This may indicate that PDAC with low stromal component is more likely to have an R1 status in pathological reports. Whereas, the result may be related with a surgical bias. Probably tumors with high stromal component are characteristic by hardness, so the surgeons tended to resect more pancreatic tissue to achieve a negative margin. However, no consensus has been reached on the prognostic impact of stromal component in patients with PDAC based on previous studies (7–9).

We also found that TSR categories could be a strong and independent prognostic factor in patients with PDAC and demonstrated that the prognostic impact of TSR was almost similar with tumor grade and perineural invasion, which are regularly included in pathological reports. Thus, the TSR would considerably improve the prognostic stratification of patients with PDAC, considering the simplified and reliable assessment methods. In our study, TSR categories could successfully stratify patients according to TNM stages I, II, and III in both the developing and validation cohorts. This may be profoundly significant to manage postoperative therapy. In addition to embracing newer strategies comprising genomics, stromal therapies, and immunotherapies, conventional approaches using chemotherapy and radiotherapy still offer considerable prospects for greater traction and synergy with evolving concepts (27). Moreover, chemotherapy resistance may be closely related to stromal component (28, 29). Stroma features that improve risk assessment have the potential to facilitate treatment, leading to a more efficient management of this patient population. Considering that recent studies have shown no molecular differences between very long-term and short-term survivors among patients with PDAC (30) and that pathological prognostic markers, such as the TSR, could aid in identifying high-risk groups, TSR assessment in PDAC would be an additional factor to help select patients who would benefit from a more intensified chemotherapy approach. Thus, validated prognostic factors, including the TSR, can substantially increase the probability of a more individualized therapy and may even be added to the stage classification of tumors for better identification of patient subgroups and, consequently, for a more personalized management of patients with PDAC.

TSR assessment can be performed not only using pathology but also using radiology. Previous studies reported that the stromal component evaluated using radiology exhibited good correlation with that evaluated using pathology and high stromal component was associated with a relatively long survival time (15, 31), which coincide with the results of our research. This may be explained by the advantages of the assessment of the entire tumor, which links microscopic pathology to macroscopic radiology. More importantly, TSR calculation using radiology can predict the prognosis of metastatic tumors and may guide the management of patients undergoing neoadjuvant therapy who cannot undergo upfront surgery. In a subsequent research, we will attempt to clarify the relationship between the TSR evaluated using radiology and the prognosis of patients with PDAC, which may make TSR evaluation independent from the specimens obtained postoperatively.

The present study has several limitations. First, our study has the intrinsic shortcomings of any retrospective study. Second, the specific pathological methods for the TSR assessment used in this study made it difficult to perform external validation. We have been studying to identify the representative part of the whole tumor specimen for TSR evaluation, so that the assessment method will be pervasively applicable and external validation can be easily conducted in the future. Third, a small number of patients were evaluated using the computer-aided method.

## Conclusion

Our findings indicate that TSR evaluation in PDAC according to the assessment method that we first used and validated provides independent prognostic information complementary to the TNM staging system. Moreover, we demonstrate the robustness and potential of a simple, standardized, inexpensive, and reliable scoring system, which may facilitate routine TSR documentation in histopathology reports of patients with PDAC.

## Data Availability Statement

The raw data supporting the conclusions of this article will be made available by the authors, without undue reservation.

## Ethics Statement

The studies involving human participants were reviewed and approved by Institutional Review Board of Changhai Hospital. Written informed consent for participation was not required for this study in accordance with the national legislation and the institutional requirements.

## Author Contributions

BoL, SGu, and GJ: study idea and design. BoL, YW, HJ, BaL, XS, SGa, ZZ, and CN: acquisition, analysis, or interpretation of the data. BoL, YW, and HJ: initial drafts. SGu, JX, and GJ: critical revision for important intellectual content. All authors approved the final version of the manuscript and are accountable for all aspects of the work.

## Conflict of Interest

The authors declare that the research was conducted in the absence of any commercial or financial relationships that could be construed as a potential conflict of interest.
