# Supplementary figures and images for: Pros and Cons: High Proportion of Stromal Component Indicates Better Prognosis in Patients With Pancreatic Ductal Adenocarcinoma—A Research Based on the Evaluation of Whole-Mount Histological Slides
Source: Front Oncol. 2020 Aug 21;10:1472. doi: 10.3389/fonc.2020.01472 (PMC7471248; doi:10.3389/fonc.2020.01472)

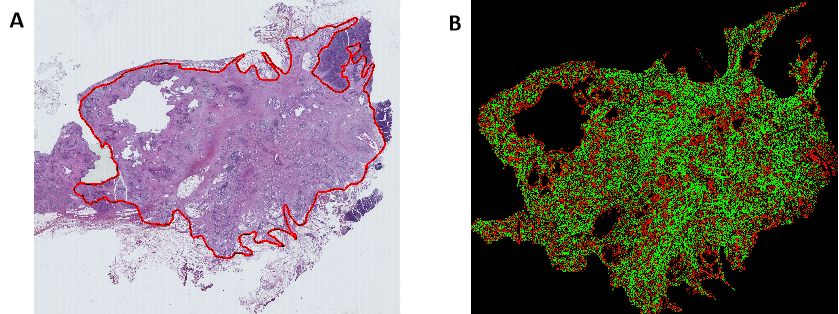

Supplement: Supplementary file 1 [file Image_1.JPEG]
